# Dermatomal somatosensory evoked potentials and cortical somatosensory evoked potentials assessment in congenital scoliosis

**DOI:** 10.1186/s12883-022-02579-4

**Published:** 2022-02-15

**Authors:** Zhenxing Zhang, Yi Wang, Tao Luo, Huaguang Qi, Lin Cai, Yang Yuan, Jingfeng Li

**Affiliations:** 1 Department of neurospinal, Xi’an HongHui Hospital Affiliated of Xi’an Jiaotong University, Xi’an, Shaanxi China; 2 Department of Orthopedics, Zhongnan Hospital of Wuhan University, Wuhan, Hubei China; 3 Department of Functional Examinantion, Xi’an HongHui Hospital Affiliated of Xi’an Jiaotong University, Xi’an, Shaanxi China

**Keywords:** Congenital scoliosis, Dermatomal somatosensory evoked potentials (DSEPs), Cortical somatosensory evoked potentials (SSEPs), Sensitivity

## Abstract

**Background:**

The aim of this study was to assess the value of dermatomal somatosensory evoked potentials (DSEPs) and cortical somatosensory evoked potentials (SSEPs) in monitoring spinal cord function for patients with congenital scoliosis (CS).

**Methods:**

This retrospective study reviewed the medical records of patients (*n* = 102) who underwent DSEP (T2-S1 dermatome), of whom 60 were normal subjects and 62 with congenital scoliosis. The study analyzed the latencies and peaks of N1-L, N1-R, P1-L and P1-R recorded by DSEPs of patients’ thoracolumbar dermatomes. To observe the incidence of abnormal DSEPs and SSEPs in CS patients and to analyze the difference in sensitivity and reliability between the two in the examination of scoliosis patients. SPSS 22.0 statistical software package was used to analyze the data, and χ2 test and correlation analysis were used to indicate that the difference was statistically significant, *p* < 0.05.

**Results:**

Sixty two patients with CS were evaluated with total spine magnetic resonance imaging (MRI). Only 23 patients (37.09%) showed spinal cord malformations in the MRI findings. The DSEP recordings showed a relatively high sensitivity (97.8%) compared to the abnormality rate of SSEPs recordings, and the rates of waveform, latency and amplitude abnormalities were much higher in DSEPs recordings (36.6, 36.3, 24.8%) than in SSEPs recordings (3.2, 22.5, 14.5%). The abnormality rate of DSEP records with and without neurological symptoms was higher than the abnormality rate of SSEP records (100% vs 20, 96.2% vs 44.2%, *p*<0.05). And in 62 patients with CS, the rate of positive MRI (37.1%) was lower than that recorded by DSEP (79.6% / 57.9%). *p* < 0.05.

**Conclusion:**

DSEPs are more sensitive to microscopic posterior column dysfunction in patients with CS that cannot be detected by either radiology or routine clinical examination. Preoperative DSEPs assessment is recommended as a baseline examination for intraoperative monitoring and comparison with the postoperative situation. DSEPs recording complements the information obtained from routine clinical and radiological evaluation.

## Background

Spinal deformities are caused by the formation of abnormal vertebrae. Hemivertebrae, loss of vertebrae or intervertebral union can cause asymmetric growth, resulting in secondary deformities [1]. Patients with congenital scoliosis often have a combination of spinal cord developmental malformations (including spinal cord embolism, spinal cord longitudinal fracture, and spinal cord cavity), the incidence of which has been reported to vary widely (18–58%) [2, 3]. Spinal cord deformities are more insidious than spinal deformities. It is difficult to detect clinically on physical examination and X-ray, and often requires Magnetic resonance imaging (MRI) and three-dimensional CT (3D-CT). MRI can show the morphological abnormalities of spinal cord malformations, and 3D CT can clearly show the vertebral bodies with developmental malformations, but both of them lack the evaluation of spinal cord nerve function [4]. X-ray / CT / MRI can only visualize morphological abnormalities of the spine and spinal cord from an imaging perspective and cannot assess the functional status of the spinal cord. Neurological examination is the best way to document the level of spinal cord injury (SCI) [5]. Preoperative assessment of the actual condition of the spinal cord injury can help to develop better interventions with the expectation of better functional recovery.

Somatosensory evoked potential (SEP) has been promoted for clinical use as a noninvasive and well tolerated technique [5]. SEP can complement the diagnostic process when the combination of clinical physical examination and spinal MRI does not identify a CS patient with a combined spinal cord injury [6]. Neurologic monitoring methods such as dermatomial somatosensory evoked potential (DSEP) and mixed somatosensory evoked potential (M-SSEP) have been developed for the early detection of nerve root injury [5]. Cortical somatosensory evoked potentials (SSEPs) records can help determine the existence, severity and prognosis of neurological deficits, as well as the functional correlation of spinal anatomic lesions. Posterior tibial somatosensory evoked potentials (PTN-SSEP) often fail to detect individual nerve root dysfunction [7]. SSEPs records were only occasionally helpful, and many false negative results were produced due to low sensitivity of standard outcome indicators [8, 9].

Dermatomial somatosensory evoked potentials (DSEPs) are recorded by stimulating the cutaneous region innervated by nerve roots, causing the excitation of peripheral nerves to the spinal cord and brain stem, and crossing the thalamus to the cortical sensory area of the brain, and corresponding waveform of cortical sensory area can be recorded on the scalp [10]. DSEPs recordings provide neurophysiological readings similar to SSEPs recordings and have been used to assess abnormalities in somatosensory pathways [11]. DSEPs objectively reflect spinal cord conduction function at any level and are used to monitor changes in sensory function in various segments after cervical medullary injury [12]. It is highly sensitive to symptoms of radiculopathy [13] and has been proven to be used for the diagnosis of neurogenic cervical spondylosis and lumbar spondylosis combined with spinal cord injury [14]. Recent studies have investigated the relationship between DSEP and lumbar spinal stenosis (LSS) [15]. However, the value of DESPs in determining spinal cord injury levels remains controversial [16].

Few studies have analyzed the utility of DSEP recordings in assessing the level of SCI in patients with CS. This study is the first to use DSEP to assess the level of spinal cord injury in patients with congenital scoliosis. The sensitivity of DSEP recordings at spinal cord functional impairment in CS patients was analyzed, and the common types of waveform alterations at different spinal cord injury levels were summarized. It provides a scientific basis for the use of DSEP examination for assessing the actual SCI and for developing targeted interventions.

## Methods

We collected and retrospectively analyzed data from the medical records of 62 patients with CS and 60 normal subjects in the neurospinal unit of Honghui Hospital Affiliated of Xi’an Jiaotong University from May 2009 to March 2020. Sixty-two CS patients, 28 males and 34 females, had a mean age of 15.9 years (6–40 years) and a mean Cobb angle of 60.2° (41°- 92°). Among them, 23 cases of scoliosis patients were complicated with spinal cord malformations (including tethered cord, diastematomyelia, syringomyelia, etc.). In addition, records of DSEPs and SSEPs from 60 normal subjects were collected as controls. All subjects had no clinically detectable neurological deficits and no history of trauma or surgery to the brain, spine, or lower extremities. This retrospective study was approved by the institutional ethics committee of Honghui Hospital Affiliated of Xi’an Jiaotong University (Approval number: 202111009).

The relevant assays for DSEPs and SSEPs were performed using a Nihon Kohden electromyography device (DANTEC MEB-9404C, Nihon Kohden, Japan). DSEP can be detected from the bottom up according to the location of the S1 dermatome (as in Tables 1 and 2) until the normal segment is detected. SSEP stimulates and records only one mixed nerve - the posterior tibial nerve. The neurophysiological examinations of all patients were completed with reference to the DSEPs and SSEPs procedures mentioned in the Slimp’ report [17]. All patients were examined preoperatively in a shielded room at 22–28 °C. A somatosensory evoked potential procedure was used, with surface electrode stimulation at 2–3 times the sensory threshold and a stimulation frequency of 3 Hz, and the signal was superimposed an average of 100 times and repeated 2 times. The stimulation sites and recording sites for DSEPs and SSEPs are shown in Tables 1 and 2, respectively. All electrophysiological recordings were quality controlled and assessed by the same training methods.

**Table 1:** SSEPs stimulation and recording site

Represents nerve roots	Stimulation site	Recording site International EEG 10-20 System Electrode Placement Method
C6—T1	Median nerve of the wrist	C3’C4’—Fz
C8—T1	Ulnar nerve of the wrist	C3’C4’—Fz
L4—S2	Tibial nerve of the medial ankle	Cz’ — Fz

**Table 2:** DSEPs stimulation and recording site

Represents nerve roots	Stimulation site	Recording site International EEG 10-20 System Electrode Placement Method
C5	5cm below the shoulder peak	C3’C4’—Fz
C6	Thumb	C3’C4’—Fz
C7	Middle finger	C3’C4’—Fz
C8	Little finger	C3’C4’—Fz
T2	Chest and arm line flat axillary crease	Cz’—Fz
T4	Flat nipple, mid-axillary line	Cz’—Fz
T6	Flat glabella, mid-axillary line	Cz’—Fz
T8	Midpoint between T6 and T10, mid-axillary line	Cz’—Fz
T10	Flat umbilicus, mid-axillary line	Cz’—Fz
T12	Flat iliac crest, midaxillary line	Cz’—Fz
L2	Midpoint of the groin	Cz’—Fz
L3	15 cm below anterior superior iliac spine	Cz’—Fz
L4	6cm medially below the inferior patellar rim	Cz’—Fz
L5	Medial aspect of the I metatarsophalangeal joint	Cz’—Fz
S1	Lateral aspect of the V metatarsophalangeal joint	Cz’—Fz

The judgment criteria of DSEPs and SSEPs are mainly to analyze the latency, interpeak latency, amplitude and the degree of waveform differentiation, and compare them. The waveforms of normal people are stable and basically similar, and the waveforms in lesions are poorly differentiated or even disappear. The latency (PL) of each wave, the latency difference (ILD) of bilateral P1 wave and the difference of bilateral amplitude (AMP) were measured. Based on our findings in normal subjects and each patient served as his or her own control on both latency and amplitude criteria. DSEPs / SSEPs recorded with one of the following conditions were considered abnormal [14, 17, 18]: ①Absolute latency > X + 2S; ②inter-lateral latency difference ≥ 2 ms; ③inter-lateral amplitude difference ≥ 50%; ④poor waveform differentiation or waveform disappearance.

For continuous variables, data were expressed as means (SDs); for categorical variables, data were expressed as frequencies. SPSS 22.0 statistical software package was used to analyze the data, and χ^2^ test and correlation analysis were used to indicate that the difference was statistically significant, *p* < 0.05.

## Results

The DSEPs and SSEPs collected from 60 normal subjects are recorded in Table 3. The DSEPs and SSEPs waveform abnormalities in 62 CS patients are shown in Table 4. The medical records of 62 CS patients with 584 DSEP findings (bilateral T2-S1 dermatome) were reviewed. Of the 584 dermatomal stimulation areas, 571 were detected as abnormal and 13 showed normal. The DSEP recordings showed a relatively high sensitivity (97.8%) compared to the abnormality rate of SSEPs recordings, and the rates of waveform, latency and amplitude abnormalities were much higher in DSEPs recordings (36.6, 36.3, 24.8%) than in SSEPs recordings (3.2, 22.5, 14.5%). In addition, we found that DSEPs recordings showed a relatively high rate of waveform differentiation abnormalities at the T4-T12 spinal cord level of 44.7% ~ 68.1%. The rates of abnormal latency delay (63.8%) and abnormal amplitude (27.6%) were highest in the T8 spinal cord segment. (Tables 3 and 4) These results suggest that spinal cord injury in CS patients often occurs in the T4-T12 segment.

**Table 3:** Data reference for normal subjects with DSEPs and SSEPs

	Stimulation location	N1-L(msec)	N1-R(msec)	P1-L(msec)	P1-R(msec)	N2-L(msec)	N2-R(msec)
DSEPs	T2	15.04±1.74	15.07±1.29	22.00±1.55	22.18±1.42	32.27±2.72	31.60±2.26
	T4	17.27±2.13	17.17±1.49	24.50±1.76	24.48±1.56	33.77±2.80	33.43±2.80
	T6	18.07±1.83	17.90±1.52	25.02±1.68	25.17±1.73	34.76±2.88	34.80±3.10
	T8	18.50±1.70	18.78±1.68	25.53±1.85	25.55±1.75	35.40±3.48	35.27±3.25
	T10	20.13±1.55	20.40±1.85	26.63±1.70	26.75±1.88	36.07±3.22	36.10±3.52
	T12	21.52±2.12	21.82±1.69	28.28±2.07	28.47±1.95	38.15±3.20	38.36±3.28
	L2	22.95±2.10	22.32±1.84	29.08±2.10	29.07±1.95	38.63±3.11	38.56±2.92
	L3	22.97±1.92	23.63±2.01	30.13±1.89	30.40±1.84	39.20±2.98	39.32±2.45
	L4	30.07±3.25	30.25±2.94	38.07±2.47	38.12±2.84	46.63±3.13	47.52±3.17
	L5	36.93±3.14	37.60±3.14	44.87±3.12	44.93±3.04	54.27±3.28	54.03±3.28
	S1	38.30±3.89	38.47±4.31	46.17±2.88	46.20±3.13	55.70±3.18	55.77±3.50
SSEPs	Tibial nerve (TIB)	31.18±2.61	31.63±2.44	38.95±2.04	38.85±2.18	47.33±3.75	46.87±3.80

**Table 4:** Analysis of DSEPS and SSEPs segmental abnormality rate

	Stimulation location	Number	Percentage (%)	Waveform			Latency			Amplitude			Total	Abnormal rate (%)
				L	R	Abnormal rate (%)	L	R	Abnormal rate (%)	L	R	Abnormal rate (%)		
DESP	T2	36	58.1	5	3	22.2	5	6	30.6	2	3	13.9		
	T4	36	58.1	12	12	66.7	6	7	36.1	4	3	19.4		
	T6	47	75.8	17	15	68.1	5	7	25.5	6	3	19.1		
	T8	47	75.8	9	12	44.7	14	16	63.8	5	8	27.6		
	T10	55	88.7	17	13	54.5	10	13	41.8	4	8	21.8		
	T12	55	88.7	16	12	50.9	10	15	45.5	5	8	23.6		
	L2	61	98.4	7	8	24.6	9	6	24.6	6	3	14.7		
	L3	61	98.4	13	10	37.7	11	14	40.9	3	7	16.4		
	L4	62	100	5	4	14.5	10	17	43.5	8	6	22.6		
	L5	62	100	7	8	24.2	9	10	30.6	4	4	12.9		
	S1	62	100	5	4	14.5	6	5	17.7	5	4	14.5		
Total		584		113	101	36.6	95	117	36.3	52	93	24.8	571	97.8
SSEP	Tibial nerve (TIB)	62	100	1	1	3.2	6	8	22.5	4	5	14.5	25	40.3

Of the 62 CS patients, 10 had neurological symptoms and 52 had no neurological symptoms (Table 5). The incidence of abnormalities in DSEPs and SSEPs was 96.8 and 40.3%, respectively. Of the 10 patients with neurological symptoms, 10 had abnormal DSEPs waveforms and 2 of the SSEPs waveforms were abnormal and 8 were normal. Of 52 patients with CS without neurological symptoms, 50 had abnormal DSEPs waveforms and 2 had normal DSEPs waveforms; the SSEP waveform was abnormal in 23 patients and normal in 27 patients. DSEPs with or without neurological symptoms had a high incidence of abnormalities 100 and 96.2%. There was a statistically significant difference in the incidence of waveform abnormalities in DSEPs and SSEPs with and without neurological symptoms (100% vs 20 and 96.2% vs 44.2%, *P* < 0.05) (Table 5).

**Table 5:** Analysis on abnormality rate of DSEPs and CSEPs

Neurologic symptoms (*n*=62)	DSEPs		SSEPs		*P* value
	Cases	Abnormality rate	Cases	Abnormality rate	
With (*n*=10)	10	100%	2	20%	<*0.05*
Without (*n*=52)	50	96.2%	23	44.2%	<*0.05*
Total abnormality waveforms	60	96.8%	25	40.3%	<*0.05*

Of the 62 CS patients, 23 had spinal MRI tests showing spinal cord malformations and 39 had no spinal cord malformations. Of the 23 CS patients with spinal cord malformations, a total of 192 dermatomal stimulation points were detected, 39 normal and 153 abnormal. Of the 39 patients without intradural CS lesions, a total of 295 dermatomal stimulation points were detected, 124 were normal and 171 were abnormal. DSEPs showed relatively high abnormal diagnostic sensitivity in patients with CS with or without spinal cord malformations (79.6, 57.9%). And the incidence of abnormal DSEPs was higher in patients with spinal cord malformations (79.6%) than in patients without spinal cord malformations (57.9%). The abnormality rates in both groups were statistically significant, *P* < 0.05 (See in Table 6).

**Table 6:** Comparison of dermatomes in DSEPs

Intraspinal pathologies	MRI		Dermatomes of DSEPs				*P value*
	Case (*n*=62)	Abnormality rate	Normal	Abnormal	Total	Abnormality rate	
With	23	37.1%	39	153	192	79.6%	*<0.05*
Without	39		124	171	295	57.9%	
*P value*						*<0.05*	

**Fig. 1:**
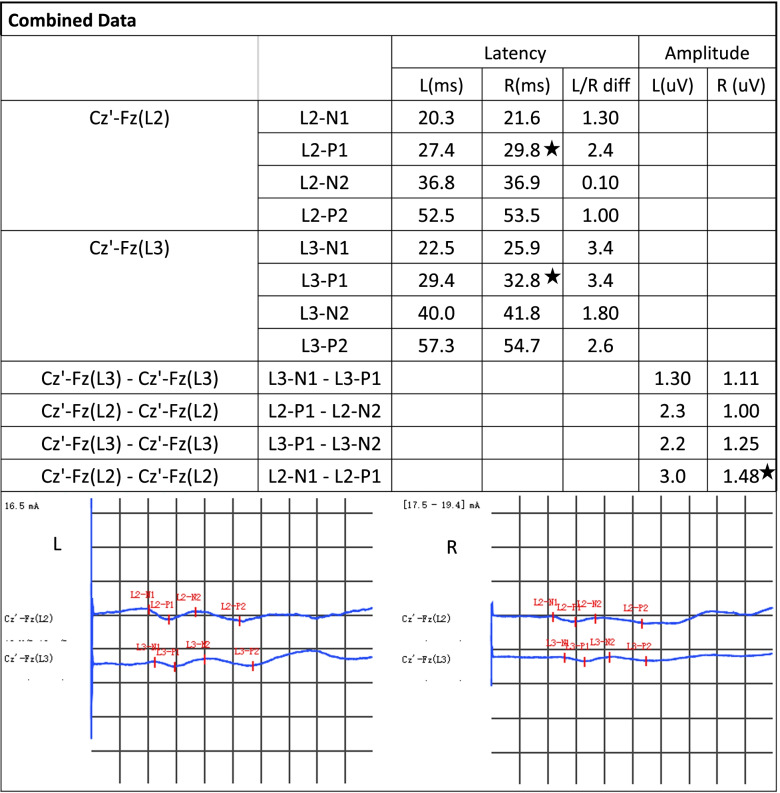
D-SEP (L2、L3):The left L2 and L3 dermatomes had normal P1 waveform latencies. The right L2 and L3 dermatomes have prolonged P1 waveform latencies. > 2 ms. **★**: Indicates prolonged incubation period

Case 1: Lumbar spine 2 and Lumbar spine3(L2 and L3)dermatomes (Fig. 1).

**Fig. 2:**
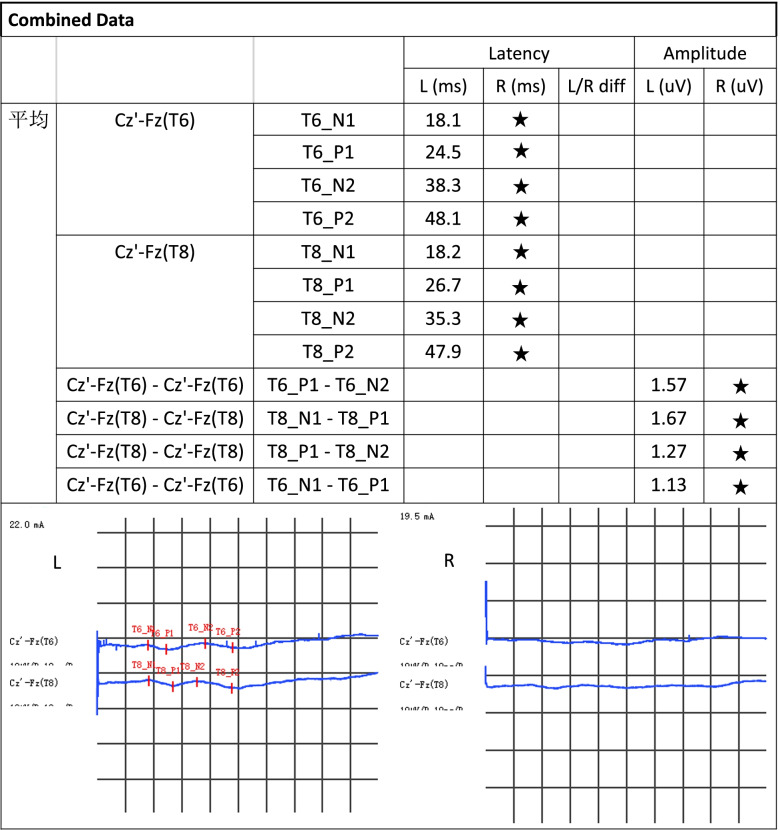
D-SEP (T6、T8): The latency of the P1 waveform was normal in the left T6 and T8 dermatomes. Poorly differentiated P1 waveforms in the right T6 and T8 dermatomes. **★:** Indicates poor waveform differentiation

Case 2:Thoracic spine 6 and Thoracic spine8 (T6 and T8) dermatomes (Fig. 2).

## Discussion

The main cause of spinal cord dysfunction in CS is compression, stretch or rotation of spinal cord secondary to the spinal deformity itself. Posterior column dysfunction in patients with CS who were are sensitive to vibration in the lower extremity compared with normal population has been demonstrated [19]. Neurophysiological methods are used to assess the functional status of the central nervous system and are important adjuncts to the clinical examination. Somatosensory evoked potentials (SEPs), electromyography (EMG), and nerve conduction studies (NCS) are commonly used to assess the function of the spinal cord and nerve roots [10]. DSEPs are used to assess the level of spinal cord injury in a single segment by stimulating the cutaneous distribution area of sensory fibers of a single posterior spinal nerve root and recording waveforms with locking time relationships in the cerebral cortex [20]. DSEPs have been reported to be useful in assessing patients with lumbosacral disease, cervical spondylosis and spinal stenosis, and in showing the segmental level of myelopathy [21–23], and also as an important indicator for the assessment of brachial plexus injury [24]. The latency and amplitude of the N33 and P40 components of the DSEP waveform were analyzed [25].

In this study, DSEPs and SSEPs were evaluated in 62 patients with congenital scoliosis using this method, and the assessment of the level of SCI is crucial because the deformity of vertebrae and nerves in patients with congenital scoliosis may cause compression and degeneration of nerve roots in the corresponding segment. The DSEPs can be detected from the bottom up according to the location of the S1 dermatome (as in Table 2) until the normal segment is detected. SSEPs reflect the conduction function of the posterior spinal cord and stimulate only one site of the posterior tibial nerve. DSEP has more fixed dermatomal stimulation sites, and poor waveform differentiation at a single stimulation site is considered abnormal in DSEP. Therefore, the sensitivity of abnormal waveform detection rate is higher than that of SSEP. In this study, DSEPs were detected in 62 patients with congenital scoliosis based on 584 locations in 11 segments from T2 to S1 at the site of deformity, and 36.6% of waveforms disappeared, 36.3% of latency abnormalities, and 24.8% of wave amplitude abnormalities; whereas in 62 patients with SSEPs detected in the posterior tibial nerve, 3.2% of waveforms disappeared, latency abnormalities 22.5, and 14.5% of the waveform amplitude abnormalities. The DSEPs recordings showed a relatively high sensitivity (97.8%) compared to the abnormal rate of SSEPs recordings. In addition, analysis of the data in Table 4 showed a significantly higher rate of abnormal waveform differentiation in the T4-T12 dermatomal region, indicating a greater probability of nerve root or spinal cord injury in the thoracic segment of congenital scoliosis, which may be related to greater compression of the nerve or spinal cord here. It is of great interest to assess nerve root function in conjunction with DSEPs results in the selection of approach, osteotomy location and orthopedic angle during surgery.

SSEPs recordings help to determine the presence, severity and prognosis of neurological deficits, as well as the functional relevance of anatomical lesions of the spinal cord. However, SSEPs responses of mixed nerves cannot be used to accurately assess the physiological status of individual nerve roots because mixed nerves enter the spinal cord from multiple levels. In this study, DSEPs and SSEPs were statistically analyzed in patients with scoliosis with or without clinical symptoms (Table 5), in which 10 patients with clinical symptoms had 100% abnormal DSEPs compared with 20% abnormal SSEPs. Fifty two patients without clinical symptoms had 96.2% abnormal DSEPs compared with 44.2% abnormal SSEPs, and all 62 CS patients had an abnormal DSEPs rate of 96.8% compared with 40.3% for SSEPs. The results suggest that abnormal DSEPs in patients with congenital scoliosis do not correlate with clinical symptoms (100% vs 96.2%). The rate of abnormal SSEPs was significantly lower in patients with neurological symptoms than in patients without neurological symptoms (20% vs 44.2%), and the results suggest that clinical neurological examination is inconsistent with SSEPs findings. Therefore, in patients with congenital scoliosis, regardless of whether the patient has clinical symptoms, DSEP and SSEP testing is recommended to better assess the functional status of the nerve roots and spinal cord (Table 5).

The incidence of congenital scoliosis with spinal cord developmental abnormalities is more common in clinical practice, and the variation in its incidence varies among reports. Blake [26] et al. reported 58% of 108 patients with congenital scoliosis with spinal cord developmental abnormalities by myelography, and they suggested that the increased rate of abnormalities was related to the diagnostic technique. MRI is still the method of choice for the diagnosis of spinal cord malformations [27]. All patients suspected of having a spinal cord deformity require MRI to confirm the diagnosis. However, MRI only reflects the morphological abnormalities of the spinal cord deformity and does not indicate whether the spinal cord deformity affects the neurological function of the patient [28], whereas SSEPs and DSEPs can reflect the neurological function of the patient. DSEPs could objectively reflect the conducting function of any level of the spinal cord and their segmental specificity is expected to be more than those of SSEPs [29]. SSEPs are electrophysiologically reported to be obtained by stimulation of the posterior tibial nerve, a mixed nerve consisting of three and more nerve roots such as L4-S2. The nerve root or spinal cord injury present in patients with congenital scoliosis is often the result of a combination of segmental demyelination and varying degrees of axonal degeneration, and electrical stimulation can lead to simultaneous excitation of multiple nerve roots, overcoming the abnormal conduction of the involved nerve roots. This affects the sensitivity of posterior tibial nerve SSEPs for electrophysiological diagnosis in patients with scoliosis. The large-scale and multisegmental advantages of DSEPs exactly compensate for the disadvantages of posterior tibial nerve SSEPs [17, 18]. In patients with congenital scoliosis, there are more spinal deformity segments and a larger range of DSEP abnormalities with different manifestations. The results in Table 6 show that the rate of MRI finding spinal deformity abnormalities was 37.1% and the rate of DSEP finding neurological abnormalities was 76.9%; in the 39 patients with no spinal deformity found by MRI, the rate of DSEPs abnormalities was 57.9%. Therefore, this study showed that the abnormal rate of DSEPs was significantly higher in patients with spinal cord deformities detected by MRI, suggesting that DSEPs testing can reflect the level of multisegmental spinal cord injury and can detect neurological impairment that cannot be diagnosed on clinical physical examination, and is an important supplement to MRI examination.

**Future and limitations:** In the future, DSEPs examination will play a more important role in preoperative assessment, intraoperative intervention and postoperative recovery as an index of spinal cord injury assessment in CS patients with concomitant spinal cord injury. Although the evoked potential amplitude of a single nerve root in certain dermatomal regions is greater than that of adjacent nerve roots, stimulation of this region is considered equivalent to activation of a single dermatomal region. However, specific cutaneous areas are rarely innervated by a single spinal nerve and often overlap with adjacent cortical areas. At the same time, there is no systematic comparison with existing neurophysiological detection techniques. These have led to some controversy regarding DSEP in the diagnosis of spinal nerve injury.

## Conclusion

In summary, DSEPs are more sensitive than SSEPs in detecting subtle posterior column dysfunction in patients with congenital scoliosis, which is not detectable on radiological and routine clinical examinations. Preoperative DSEP assessment can be used as a baseline examination for intraoperative monitoring, which helps the surgeon to adjust the surgical treatment plan in a timely manner and facilitates the assessment of postoperative neurological recovery status. DSEP examination is an important complement to conventional clinical physical examination and imaging assessment as a means of assessing nerve root and spinal cord function.

## Data Availability

The data that support the findings of this study are available from Xi’an Honghui Hospital, but restrictions apply to the availability of these data, which were used under license for the current study, and so are not publicly available. Data are however available from Yang Yuan upon reasonable request and with permission of Xi’an Honghui Hospital.

## Abbreviations

SEPs: Somatosensory evoked potentials
DSEPs: Dermatomal somatosensory evoked potentials
CSEPs: Cortical somatosensory evoked potentials
CS: Congenital scoliosis
MRI: Magnetic resonance imaging
SCI: Spinal Cord Injury
